# Supplementary material for: Low Zinc Levels at Admission Associates with Poor Clinical Outcomes in SARS-CoV-2 Infection
Source: Nutrients. 2021 Feb 9;13(2):562. doi: 10.3390/nu13020562 (PMC7914437; doi:10.3390/nu13020562)
Supplement: Supplementary file 1 [file nutrients-13-00562-s001.pdf]

Table 2A. Multivariable linear regression model studying the impact in serum zinc concentration adjusted by age, gender, severity and comorbidity (Charlson Comorbidity Index) in the time to recovery

	beta-coeff	95% CI	p-value
Zinc (μg/dl)	-0.21	(-0.356 ;0.155)	<0.001
Gender	0.52	(-2.61 ; 3.67)	0.744
Age	0.036	(-0.097 ;0.1517)	0.537
MEWS (severity score)	1.441	(.173 ; 2.708)	0.026
Charlson Comorbidity Index	0.134	(-0.499 ; 0 .767)	0.677

Table 2B. Multivariable linear regression model studying the impact in serum zinc concentration as a dichotomous variable (SZC <50 μg/dl or *≥50* μg/*/dl) adjusted by age, gender, severity and comorbidity (Charlson Comorbidity Index) in the time to recovery*

	beta-coeff	95% CI	p-value
Zinc <50ug/dl	14.1	(90.23;17.07)	<0.001
Gender	-0.291	(-3.34; 2.75)	0.851
Age	0.07	(-0.039 ; 0.181)	0.209
MEWS (severity score)	1.747	(0.57;2.92)	0.004
Charlson Comorbidity Index	0.151	(-0.454 ; 0 .759)	0.623
